# On the etiology of tropical epidemic neuropathies.

**DOI:** 10.3201/eid0502.990231

**Published:** 1999

**Authors:** J de la Fuente, M P Rodríguez


Dr. Matteson claims that DDT is associated with reduced lactation. In the United States, where DDT has been banned for 26 years, mothers who stay home breast-feed for an average of 25.1 weeks—mothers who work parttime, for 22.5 weeks (19). In Belize, mothers in urban areas, where DDT is not used for malaria control, breast-feed less than 38.4 weeks—mothers in rural areas with lifetime exposures to DDT breast-feed more than 57.2 weeks (20).

The World Wildlife Fund's mass balance model of DDT sprayed in houses used to refute our assessment that DDT does not readily move away from sprayed houses also mentions that "There are few...data against which to validate the results of this...model, although actual data...should not be difficult to obtain." (21). Studies of DDT use in agriculture show that most DDT settles where it is applied (22).

Studies have shown no meaningful population-based adverse health effects from DDT use, despite more than 50 years' exposure, and evidence argues forcefully that DDT does not cause breast cancer (23).

**Donald R. Roberts and Larry L. Laughlin**
The Uniformed Services University of the Health Sciences, Bethesda, Maryland, USA

### References

1. Roberts DR, Laughlin LL, Hsheih P, Legters LJ. DDT, global strategies, and a malaria control crisis in South America. Emerg Infect Dis 1997;3:295-302.
2. Brasil. Registro de casos de malária—1960 a 1997. Gerência Técnica de Malária/FNS-Brasilia, Brasilia, Brasil.
3. Pan American Health Organization. Status of malaria programs in the Americas. XL report. Washington: The Organization; 1991. p. 145.
4. Pan American Health Organization. Status of malaria programs in the Americas. XLII report. Washington: The Organization; 1994. p. 116.
5. Pan American Health Organization. Status of malaria programs in the Americas. XLIII report. Washington: The Organization; 1995. p. 25.
6. Pan American Health Organization. Status of malaria programs in the Americas. XLIV report. Washington: The Organization; 1996. p. 23.
7. Roberts DR. Resurgent malaria: DDT and global control. U.S. Medicine 1998;34:36-8.
8. de Bustamante FM. Distribuição geográfica e periodicidade estacional da malária no Brasil e sua relação com os fatores climáticos. Situação atual do problema. Revista Brasileira de Malariologia e Doenças Tropicais 1957;9:181-90.
9. Pinheiro FP, Bensabath G, Rosa APAT, Lainson R, Shaw JJ, Ward R, et al. Public health hazards among workers along the Trans-Amazon Highway. Journal of Occupational Medicine 1977;19:490-6.
10. Smith NJH. Colonization lessons from a tropical forest. Science 1982;13:755.
11. Roberts DR. Health problems of colonists. Science 1982;217:484.
12. Sandoval JJF, Diniz R, Saraiva MGG, da Silva EB, Alecrim WD, Alecrim MGC, et al. Histórico da malária na cidade de Manaus e proposta de controle integrado. Rev Soc Bras Med Trop 1998;31, Suplemento 1:141.
13. Amaral JCOF, Machado RLD, Segura MNO, Oliveira GS, Povoa MM. Avaliação longitudinal da infecção causada por *Plasmodium falciparum* e/ou *Plasmodium vivax* na população de duas localidades de Icoaraci, Distrito de Belem, Para. Rev Soc Bras Med Trop 1998;31 Suplemento 1:16.
14. da Silva EB, Costa MF, Melo YFC, Alecrim MGC. Inquérito soroepidemiologico numa área urbana em fase de ocupação, na cidade de Novo Aryao-Amazonas-Brasil. Rev Soc Bras Med Trop 1998;31 Suplemento 1:82.
15. Ventura AM, Pinto AY, Uchoa R, Calvosa V, Santos MA, Filho MS, et al. Malária por *Plasmodium vivax* em crianças-I-aspectos epidemiologicos e clínicos. Rev Soc Bras Med Trop 1998;31 Suplemento 1:82.
16. Suarez MC, Fe NF, Alecrim WD. Estudo do processo de transmissão da malária em uma área de invasão recente na cidade de Manaus Amazonas. Estudo entomologico. Rev Soc Bras Med Trop 1998;31 Suplemento 1:15-6.
17. Forattini OP. Entomologia medica: I volume parte Geral, Diptera, Anophelini. São Paulo (Brasil): Faculdade de Higiene e Saúde Publica; 1962. p. 414.
18. Rachou RG. Some manifestations on behaviouristic resistance in Brazil. Semina Suscep. Insects to insecticides, Panama, Report.: WHO 1958:208-95.
19. Frank E. Breastfeeding and maternal employment: two rights don't make a wrong. Lancet 1998;352:1083-4.
20. Central Statistical Office, Belize. 1991 Belize family health survey, final report. Reprinted by U.S. Dept of Health and Human Services; 1992. p. 69.
21. Resolving the DDT dilemma: protecting biodiversity and human health. Toronto, Canada: World Wildlife Fund-Canada; 1998.
22. World Health Organization. DDT and its derivatives. Environmental health criteria 9. Geneva: The Organization; 1979. p. 194.
23. Safe, SH. Xenoestrogens and breast cancer. N Engl J Med 1997;337:1303-4.

### On the Etiology of Tropical Epidemic Neuropathies

**To the Editor:** In a recent report of an epidemic of optic neuropathy in Dar es Salaam, Tanzania (1), Dolin et al. state that the disease is clinically identical to one of the forms of epidemic neuropathy found in Cuba between 1991 and

1993 (2). Cases of peripheral neuropathy have been part of both epidemics (1,2). Both epidemics occurred in nutritionally deficient populations (1,3).

Dolin et al. state that the cause of the Tanzanian epidemic is unknown and probably difficult to establish; however, we believe findings from the Cuban epidemic could be used to study the etiology of this and other tropical epidemic neuropathies.

In Cuba, several research groups isolated and characterized an enterovirus in the cerebrospinal fluid (CSF) of epidemic neuropathy patients (4,5). Enterovirus sequences were found in CSF of 40 (36%) of 111 epidemic neuropathy patients versus 1 (8%) of 12 control surgical patients ($p < 0.01$, chi-square test with 2 x 2 contingency tables) (5). Recently, this enterovirus has been shown to form quasispecies, which could account for altered biologic properties (de la Fuente et al., submitted for pub.). We thus propose that epidemic neuropathy has a nutroviral etiology: Nutritional deficits and stress make the population more likely to become ill after infection with enterovirus quasispecies with altered biologic properties.

The relationship between the host's nutritional status and virus evolution could be key in understanding the cause of epidemic neuropathy, the Tanzanian epidemic of optic neuropathy, and other tropical epidemic neuropathies. Etiologic factors must be identified before appropriate intervention and treatment strategies can be implemented.

**José de la Fuente and María P. Rodríguez**
Centro de Ingeniería Genética y Biotecnología,
Havana, Cuba

### References

1. Dolin PJ, Mohamed AA, Plant GT. Epidemic of bilateral optic neuropathy in Dar es Salaam, Tanzania. N Engl J Med 1998;338:1547-8.
2. The Cuba Neuropathy Field Investigation Team. Epidemic optic neuropathy in Cuba—clinical characterization and risk factors. N Engl J Med 1995;333:1176-82.
3. Román GC. On politics and health: an epidemic of neurologic disease in Cuba. Ann Int Med 1995;122:530-3.
4. Más P, Pelegrino JL, Guzmän MG, Comellas MM, Resik S, Alvarez M, et al. Viral isolation from cases of epidemic neuropathy in Cuba. Arch Pathol Lab Med 1997;121:825-33.
5. Rodríguez MP, Alvarez R, García del Barco D, Falcón V, de la Rosa MC, de la Fuente J. Characterization of virus isolated from the cerebrospinal fluid of patients with epidemic neuropathy. Ann Trop Med Parasitol 1998;92:97-105.

### Risk for Ebola Virus Infection in Côte d'Ivoire

**To the Editor:** In Taï National Park, Côte d'Ivoire, where a new strain of Ebola virus was isolated (1), the World Health Organization is conducting a project to identify the reservoir of the virus and evaluate the risk for its emergence in local populations. In March 1998, we conducted qualitative and quantitative surveys of the villagers' awareness of and risk for Ebola infection. In four villages close to Taï National Park (4 km to 10 km), we carried out structured interviews with 150 villagers and in-depth interviews with 17 villagers and three traditional healers.

Of the 150 villagers participating in the structured interviews, 18.0% had heard of Ebola (90.7% had heard of yellow fever). Of those aware of Ebola, 96.3% thought it life-threatening; 65.4% of them thought it preventable. When ill, 81.2% of the respondents generally relied on traditional healers or herbal medicine. During in-depth interviews traditional healers discussed their treatment practices. In one treatment, an incision is made on the skin and medicinal herbs are applied to the incision. Such traditional practices were implicated in the spread of Ebola virus in Gabon, where a traditional healer and his assistant (who were infected with Ebola virus) were suspected of spreading the virus to their patients through an unsterilized blade (1). The same practices would seem to pose a risk for virus transmission in Côte d'Ivoire.

Even though officially Taï National Park is protected from human activities to preserve its natural ecology, 84.0% of the 150 respondents to our survey often hunted or farmed in the park, 62.2% had encountered chimpanzees, and 53.3% had eaten chimpanzee meat. According to the in-depth interviews, chimpanzee meat is available at bush meat markets and is thought safe for eating, even though primates infected with Ebola virus have been linked with human cases (2,3).

